# Right ventricular free wall strain predicts quality of life in repaired tetralogy of Fallot

**DOI:** 10.1186/1532-429X-15-S1-E85

**Published:** 2013-01-30

**Authors:** Jimmy C Lu, Maryam Ghadimi Mahani, Prachi Agarwal, Adam L Dorfman

**Affiliations:** 1 Pediatrics and Communicable Diseases, Divsion of Pediatric Cardiology, University of Michigan, Ann Arbor, MI, USA; 2 Radiology, University of Michigan, Ann Arbor, MI, USA

## Background

After repair of tetralogy of Fallot (rTOF), left (LVEF) and right (RVEF) ventricular ejection fraction are important markers of clinical status and outcome, but may not improve after pulmonary valve replacement. Strain is a sensitive marker of ventricular dysfunction, but the potential relation to quality of life in this population has not been evaluated.

## Methods

Fifty-nine patients with rTOF (median age 28 years, range 15-69) underwent cardiovascular magnetic resonance (CMR) from 2008-2009 and completed the Short Form 36 (a validated quality of life assessment). A midventricular short-axis slice was analyzed for left ventricular global circumferential strain (LVGCS) with feature tracking software (TomTec, Unterschleissheim, Germany). A four-chamber slice was analyzed for left ventricular longitudinal strain (LVGLS), and right ventricular longitudinal strain (RVLS), using the three segments of the right ventricular free wall. A subset of 30 patients was re-analyzed for intraobserver and interobserver variability. The physical component summary (PCS) and subscales of physical functioning (PF), role-physical (RP) and general health (GH) were chosen for analysis, and age-adjusted z-score ≤-1 was considered a clinically significant decreased quality of life.

## Results

Strain analysis was feasible in 58/59 patients (98%). LVGCS correlated with LVGLS (r=0.45, p=0.006) and LVEF (r= -0.63, p<0.0001). RVLS correlated with LVEF (r= -0.29, p=0.03) but not with RVEF. Patients with RVLS below the median had increased odds of decreased PF and GH subscale scores, with strong trends for PCS and RP (Table). Among patients with normal RVEF (≥45%) these associations remained significant (PF: OR 9.5, p=0.03; GH: OR 5.9, p= 0.04). Intraobserver and interobserver variability was acceptable for LVGCS (coefficients of variation 9.5%, 10.0%), but lower for LVGLS (17.2%, 16.8%), and poor for RVLS (19.9%, 28.8%).

**Table 1:** Odds ratios of age-adjusted z-score ≤-1 with strain value below the median. Data presented as odds ratio (95% confidence interval).

	PCS	PF	RP	GH
LVGCS	1.7 (0.5-6.1) p= 0.39	2.8 (0.8-12) p= 0.13	2.7 (0.6-14) p= 0.19	1.7 (0.5-5.5) p= 0.40

LVGLS	1.7 (0.5-6.1) p= 0.39	1.2 (0.3-4.5) p= 0.76	0.6 (0.1-2.6) p= 0.51	1.2 (0.4-3.8) p= 0.78

RVLS	2.1 (0.6-7.3) p= 0.12	5.4 (1.4-27) p= 0.01	3.4 (0.8-18) p= 0.10	3.5 (1.1-13) p= 0.04

## Conclusions

Although RVLS reflects right ventricular sinus function and not necessarily overall RVEF, it appears to have discriminative ability in this population for decreased quality of life, and may yield incremental prognostic value beyond global RVEF assessment. However, clinical use may be limited by reproducibility. Further study is needed to evaluate techniques to limit variability, and to evaluate longitudinal changes in this population.

## Funding

The authors have nothing to disclose.

